# Longitudinal Dynamics of NK-Cell Regulatory Signaling and IVIG Response in Kawasaki Disease

**DOI:** 10.3390/children13050635

**Published:** 2026-05-02

**Authors:** Yeonju Kim, Insu Choi, Kyung Soon Choi, In Seok Jeong, Hwa Jin Cho

**Affiliations:** 1 Department of Pediatrics, Chonnam National University Medical School, Chonnam National University Children’s Hospital, Gwangju 61469, Republic of Korea; yjsky1203@naver.com (Y.K.); realalice@hanmail.net (I.C.); 2 Extracorporeal Cardiopulmonary Innovation, Technology and Education (EXCITE) Research Group, Chonnam National University Hospital, Gwangju 61469, Republic of Korea; queen050178@naver.com; 3 Department of Thoracic and Cardiovascular Surgery, Chonnam National University Hospital and Medical School, Gwangju 61469, Republic of Korea

**Keywords:** Kawasaki disease, natural killer cells, IVIG resistance, NK-cell regulatory receptors, innate immune regulation

## Abstract

**Highlights:**

**What are the main findings?**

- Baseline NK-cell regulatory receptor expression (NKG2D/NKG2A ratio) was not associated with IVIG resistance in Kawasaki disease.
- Longitudinal changes in NK-cell receptor expression differed according to IVIG response, suggesting dynamic immune modulation after treatment.

**What is the implication of the main finding?**

- Baseline immune profiling alone may be insufficient to predict IVIG resistance, highlighting the importance of longitudinal immune monitoring.
- Dynamic changes in NK-cell regulatory pathways may provide insights into disease mechanisms and potential therapeutic targets in Kawasaki disease.

**Abstract:**

*Background and Objectives*: Kawasaki disease (KD) is an acute systemic vasculitis in children, and approximately 10–20% of patients develop resistance to intravenous immunoglobulin (IVIG), which is associated with an increased risk of coronary artery complications. Natural killer (NK) cells play an important role in innate immune regulation, but the temporal dynamics of NK-cell regulatory receptors during KD and their relationship with IVIG response remain unclear. *Materials and Methods*: In this prospective observational study, we performed longitudinal immunophenotyping in children with KD treated at a tertiary referral center. Peripheral blood samples were obtained before IVIG administration (D0) and at three follow-up timepoints after treatment (D2, D14, and D56). NK-cell subsets and receptor expression—including the activating receptor NKG2D and inhibitory receptor NKG2A—were analyzed using multiparameter flow cytometry. Associations with IVIG response were evaluated using Firth penalized logistic regression for baseline predictors and linear mixed-effects models to assess longitudinal immune trajectories. *Results*: A total of 69 patients with KD were included, of whom 17 (24.6%) were classified as IVIG resistant. Baseline NK-cell subsets and receptor expression did not differ significantly between IVIG-sensitive and IVIG-resistant patients, although the NKG2D/NKG2A ratio tended to be lower in resistant patients (median 2.51 vs. 3.34, *p* = 0.054). Longitudinal mixed-effects analysis demonstrated significant temporal changes in NK-cell regulatory signaling following IVIG therapy. Both NKG2A (P(time) = 0.019) and NKG2D (P(time) < 0.001) expression showed significant time effects across the disease course. Importantly, the NKG2D/NKG2A ratio demonstrated a significant time-by-group interaction (P(interaction) = 0.030), indicating divergent trajectories of activating and inhibitory NK-cell signaling according to IVIG response. At the convalescent phase (D56), IVIG-resistant patients showed significantly higher NKG2A expression (*p* = 0.038) and a lower NKG2D/NKG2A ratio (*p* = 0.023) than IVIG-sensitive patients. *Conclusions*: While baseline NK-cell immunophenotypes were not associated with IVIG response, longitudinal analysis revealed that IVIG-resistant patients exhibited a distinct immune trajectory, characterized by increased NKG2A expression and a lower NKG2D/NKG2A ratio during the convalescent phase. These findings suggest that differences in IVIG responsiveness may be related to alterations in immune regulatory processes during the resolution phase of inflammation. However, the clinical implications of these findings remain to be established and require validation in larger, multicenter studies with longitudinal outcome data.

## 1. Introduction

Kawasaki disease (KD) is an acute systemic vasculitis of childhood and remains the leading cause of acquired heart disease in children in developed countries [1]. Although treatment with high-dose intravenous immunoglobulin (IVIG) significantly reduces the risk of coronary artery complications, approximately 10–20% of patients develop IVIG resistance, which is associated with a higher risk of persistent inflammation and coronary artery abnormalities [1,2]. Identifying immunologic mechanisms associated with treatment response remains an important unmet need in KD.

Natural killer (NK) cells play an important role in innate immune responses and immune regulation [3]. Previous immune profiling studies have suggested that alterations in NK-cell subsets may be associated with IVIG responsiveness in Kawasaki disease. In particular, alterations in specific NK-cell populations have been reported in IVIG-resistant patients, suggesting dysregulation of NK-cell activation pathways [4,5].

NK-cell activity is tightly regulated by the balance between activating and inhibitory receptors. Among these, NKG2D is a major activating receptor that promotes NK-cell cytotoxicity and cytokine production, whereas NKG2A functions as an inhibitory receptor that limits NK-cell activation [6,7]. The functional state of NK cells is therefore determined not only by individual receptor expression but also by the balance between activating and inhibitory signals [8].

Although immune dysregulation involving both innate and adaptive immune pathways has been described in KD, the temporal dynamics of NK-cell regulatory receptors during the course of the disease remain poorly understood [9]. In particular, whether early changes in NK-cell activating and inhibitory receptor expression are associated with IVIG responsiveness has not been clearly defined.

Therefore, in this study we performed longitudinal immunophenotyping of NK cells in patients with KD at multiple time points from the acute to convalescent phases. We aimed to characterize the temporal dynamics of NK-cell regulatory receptors, focusing on NKG2A, NKG2D, and their relative balance (NKG2D/NKG2A ratio), and to evaluate their association with IVIG treatment response.

## 2. Materials and Methods

### 2.1. Study Design and Participants

This prospective observational study was conducted at a single tertiary referral center. Children diagnosed with KD and admitted to Chonnam National University Hospital between 1 January 2023 and 31 December 2025 were screened for eligibility. The study protocol was approved by the Institutional Review Board of Chonnam National University Hospital (CNUH-2017-257), and written informed consent was obtained from the parents or legal guardians of all participants prior to enrollment. The study was conducted in accordance with the Declaration of Helsinki. Patients transferred from other hospitals after receiving the first dose of intravenous immunoglobulin (IVIG) were excluded to ensure consistent baseline sampling prior to treatment initiation.

All patients were treated according to the American Heart Association guidelines for KD. Standard initial therapy consisted of IVIG at a dose of 2 g/kg administered as a single infusion, together with aspirin at 30–50 mg/kg/day during the acute febrile phase, which was subsequently reduced to 3–5 mg/kg/day after defervescence. IVIG resistance was defined as persistent or recrudescent fever at least 36 h after completion of the first IVIG infusion. Patients meeting this definition received a second IVIG infusion at the same dose. If fever persisted following the second IVIG infusion, adjunctive therapy with intravenous methylprednisolone pulse therapy (30 mg/kg/day for 3 days) was administered. In cases of persistent fever after corticosteroid therapy, infliximab (5 mg/kg) was given.

Demographic and clinical data were collected prospectively, including age, sex, body weight, clinical manifestations of KD, laboratory parameters, and treatment information.

Blood Sampling and Clinical Laboratory Data.

Peripheral blood samples were collected at predefined time points: at diagnosis prior to intravenous immunoglobulin (IVIG) administration (D0), early after treatment (D2), during the subacute phase (D14), and during the convalescent phase (D56). Routine laboratory evaluations at diagnosis included complete blood count, C-reactive protein, and pro–brain natriuretic peptide levels.

### 2.2. Flow Cytometry and Immunophenotyping

Peripheral blood samples were analyzed using multiparameter flow cytometry to characterize natural killer (NK) cell subsets and receptor expression. To ensure measurement reliability, only freshly processed samples were included in the analysis. Samples with delayed processing were excluded due to the potential impact on flow cytometry results. After red blood cell lysis using ammonium chloride–based lysis buffer (BD Pharm Lyse; Becton Dickinson Biosciences, San Diego, CA, USA), leukocytes were washed with phosphate-buffered saline and processed according to standardized protocols. Data acquisition was performed using a BD FACSLyric^TM^ flow cytometer (BD Biosciences, Franklin Lakes, NJ, USA). Flow cytometry analysis was performed using a standardized sequential gating strategy. Lymphocytes were first identified based on forward and side scatter properties, followed by exclusion of doublets. NK cells were defined as CD3−CD56+ cells. Receptor expression (NKG2D and NKG2A) was assessed within the gated NK-cell population. Positive and negative populations were defined using fluorescence-minus-one (FMO) controls to determine threshold boundaries. Receptor expression was reported as the percentage of positive cells (Gate %).

NK-cell populations were identified based on CD56 and CD16 expression. NK-cell subsets were classified as CD56brightCD16+/−, CD56+CD16−, CD56+CD16+, and CD56−CD16+ populations. Expression of activating and inhibitory NK-cell receptors was quantified within the gated NK-cell population, including NKG2D (CD314), NKG2A (CD159a), NKp46 (CD335), and KIR2DL1 (CD158a). Receptor expression was reported as the percentage of positive cells (Gate %). The NKG2D/NKG2A ratio was calculated using percentage values. Additional lymphocyte subsets analyzed included γδ T cells (CD3+TCRγδ+) and regulatory T cells (CD4+CD25+Foxp3+).

**Figure 1:**
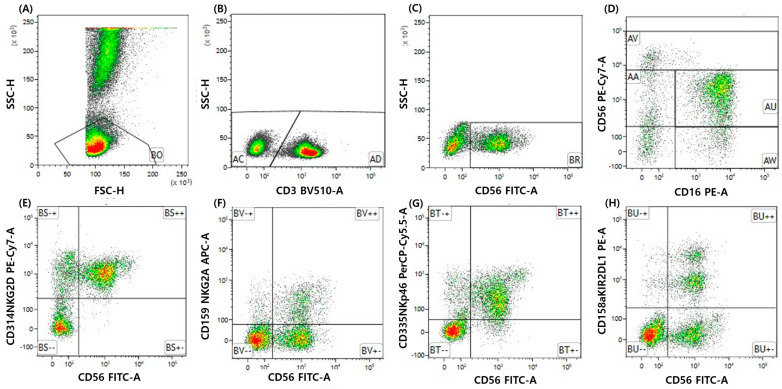
Flow cytometry gating strategy and representative NK-cell receptor expression. (**A**–**C**) Sequential gating strategy used to identify NK cells. Lymphocytes were first gated based on forward and side scatter properties, followed by exclusion of CD3+ cells and selection of CD56+ NK cells. (**D**) NK-cell subsets were defined based on CD56 and CD16 expression. (**E**–**H**) Representative flow cytometry plots showing expression of NK-cell receptors (NKG2A and NKG2D) in patients with Kawasaki disease.

Flow cytometry analyses were performed using predefined gating strategies that were applied consistently across all samples. All analyses were conducted blinded to clinical outcomes. The full antibody panel and detailed gating strategies are provided in Figure 1 and Supplementary Table S1.

### 2.3. Statistical Analysis

Continuous variables are presented as median and interquartile range (IQR), and categorical variables are summarized as counts and percentages. Baseline characteristics between IVIG-sensitive and IVIG-resistant patients were compared using the Mann–Whitney U test for continuous variables and Fisher exact test for categorical variables.

To evaluate baseline immunophenotype predictors of IVIG resistance, Firth penalized logistic regression was performed to account for the relatively small number of resistant cases. Odds ratios (ORs) with 95% confidence intervals (CIs) were reported. Given the limited number of IVIG-resistant patients, the number of variables included in the multivariable model was intentionally restricted to avoid overfitting. Prespecified variables were selected based on clinical relevance and study hypothesis.

To analyze longitudinal changes in NK-cell receptor expression and NK-cell subsets over time following IVIG treatment, linear mixed-effects models with random intercepts for each patient were constructed. Fixed effects included time, IVIG response group, and the time-by-group interaction. Separate models were fitted for NKG2A, NKG2D, and the NKG2D/NKG2A ratio. These models allowed evaluation of both overall temporal changes and differences in trajectories between IVIG-sensitive and IVIG-resistant patients.

Baseline analyses were performed using available complete cases. For longitudinal analyses, linear mixed-effects models were applied, which allow inclusion of all available observations and can appropriately handle. missing data under the assumption of missing at random.

Model-based estimated marginal means with standard errors were used to visualize temporal trends. Spearman correlation analysis was used to assess associations between NK-cell receptor expression and NK-cell subset proportions at each time point. A two-sided *p* value < 0.05 was considered statistically significant. All statistical analyses were performed using R (version 4.4.1; R Foundation for Statistical Computing, Vienna, Austria) and Python (version 3.12.4; Python Software Foundation, Wilmington, DE, USA). The sample size was determined by the number of eligible patients enrolled during the study period, reflecting the prospective and exploratory nature of the study.

## 3. Results

### 3.1. Study Cohort and Sample Availability

Among all patients with KD treated during the study period, 69 patients who provided written informed consent for immunophenotyping were included in the analysis. Of these, 17 patients (24.6%) were classified as IVIG resistant. Peripheral blood samples were obtained at predefined timepoints: baseline prior to IVIG administration (D0), early post-treatment (D2), subacute phase (D14), and convalescent phase (D56). Baseline (D0) immunophenotyping was available in 57 patients (44 IVIG-sensitive and 13 IVIG-resistant). Missing baseline samples (*n* = 12) were primarily due to logistical constraints in immediate sample processing, particularly in patients admitted during nighttime hours or weekends. Although baseline blood samples were obtained, delayed processing resulted in non-fresh samples, which were excluded to ensure the reliability of flow cytometry measurements. The number of available samples at each subsequent timepoint was 62 at D2, 67 at D14, and 58 at D56.

### 3.2. Baseline Characteristics

Baseline clinical, laboratory, and treatment characteristics according to IVIG response are summarized in Table 1. There were no significant differences between the IVIG-sensitive and IVIG-resistant groups in age, sex, body weight, or the frequency of principal clinical manifestations, including rash, conjunctivitis, red lips or tongue, cervical lymphadenopathy, extremity changes, or the proportion of complete KD. Coronary dilatation at baseline was infrequent and did not differ significantly between groups. However, patients in the IVIG-resistant group had a significantly longer duration of fever prior to IVIG administration compared with those in the IVIG-sensitive group (median, 8.0 vs. 6.0 days; *p* = 0.009).

With respect to laboratory findings at diagnosis (D0), platelet counts were lower in the resistant group (median, 310 × 10^3^/μL vs. 394 × 10^3^/μL; *p* = 0.039), while the neutrophil-to-lymphocyte ratio (median, 4.14 vs. 2.60; *p* = 0.036) and C-reactive protein levels (median, 8.12 vs. 4.68 mg/dL; *p* = 0.014) were significantly higher. Markers of hepatic involvement were also more pronounced among resistant patients, including higher alanine aminotransferase (median, 104 vs. 27 U/L; *p* = 0.027) and total bilirubin levels (median, 0.58 vs. 0.35 mg/dL; *p* = 0.022). Cardiac biomarkers, including troponin-I and pro–B-type natriuretic peptide, were modestly but significantly elevated in the resistant group. Although serum albumin levels tended to be lower in the IVIG-resistant group, the difference did not reach statistical significance (*p* = 0.06).

Adjunctive anti-inflammatory therapies were used among IVIG-resistant patients. Steroid therapy was administered in 41.2% of resistant patients and in none of the sensitive patients (*p* < 0.001). Infliximab was used in 11.8% of resistant patients and in none of the sensitive patients, although this difference did not reach statistical significance (*p* = 0.058).

### 3.3. Baseline Immunophenotype at Diagnosis

Baseline immunophenotyping at diagnosis (D0), prior to IVIG administration, was available in 57 patients, including 44 IVIG-sensitive and 13 IVIG-resistant patients. The results are summarized in Table 2. There were no significant differences between the IVIG-sensitive and IVIG-resistant groups in the overall proportion of NK cells or in the distribution of NK cell subsets, including CD56brightCD16+/−, CD56+ CD16−, CD56+ CD16+, and CD56− CD16+ populations.

With respect to NK-cell receptor expression, the proportions of NKG2D- and NKG2A-positive cells did not differ significantly between groups. The NKG2D/NKG2A ratio was lower in the IVIG-resistant group compared with the IVIG-sensitive group; however, this difference did not reach statistical significance (*p* = 0.054). Expression levels of NKp46 and KIR2DL1 were also comparable between groups. Other lymphocyte subsets, including TCRγδ-positive T cells and Foxp3-positive regulatory T cells, showed no significant differences between IVIG-sensitive and IVIG-resistant patients.

In a Firth penalized logistic regression model including fever duration before IVIG and the baseline NKG2D/NKG2A ratio, fever duration remained associated with IVIG resistance, whereas the baseline ratio was not independently associated (Supplementary Tables S2 and S3).

NKp46 and KIR2DL1 were also evaluated longitudinally; however, these markers did not demonstrate consistent temporal patterns associated with IVIG response and did not provide additional discriminatory value beyond the NKG2D-to-NKG2A ratio.

### 3.4. Temporal Dynamics of NKG2A, NKG2D, and NKG2D/NKG2A Ratio

**Figure 2:**
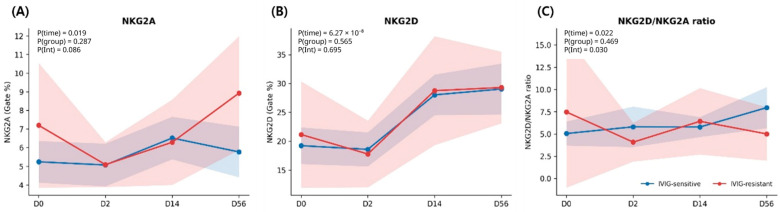
Longitudinal trajectories of NK-cell activating and inhibitory receptors according to IVIG response. Temporal changes in NK-cell receptor expression were evaluated using linear mixed-effects models with random intercepts. Estimated marginal means are shown for (**A**) NKG2A expression, (**B**) NKG2D expression, and (**C**) the NKG2D/NKG2A ratio across four study timepoints (D0, D2, D14, and D56). Shaded areas represent standard errors. Blue lines indicate IVIG-sensitive patients and red lines indicate IVIG-resistant patients. Values represent model-based estimates derived from all available longitudinal observations. These estimates may differ from raw observed values (Table 2 and Figure 3) because the mixed-effects model accounts for repeated measures and varying sample availability across timepoints. Notably, divergence in NK-cell regulatory signaling between IVIG-sensitive and IVIG-resistant patients becomes most apparent at the convalescent phase (D56), particularly in the NKG2D/NKG2A ratio.

For NKG2A, a significant overall time effect was observed (P(time) = 0.019). The main effect of the IVIG response group was not significant (P(group) = 0.287), while the time-by-group interaction showed a trend toward significance (P(interaction) = 0.087). Model-based estimated marginal means demonstrated modest increases in NKG2A expression over time in both groups, with greater variability among IVIG-resistant patients during the later phases of disease (Figure 2A).

For NKG2D, a strong overall time effect was observed (P(time) < 0.001), reflecting dynamic changes in activating receptor expression during the disease course. However, neither the main effect of the IVIG response group (P(group) = 0.565) nor the time-by-group interaction (P(interaction) = 0.695) was statistically significant, indicating similar temporal trajectories between IVIG-sensitive and IVIG-resistant patients. (Figure 2B)

When the balance between activating and inhibitory signaling was assessed using the NKG2D/NKG2A ratio, both time (P(time) = 0.022) and the time-by-group interaction (P(interaction) = 0.030) were statistically significant, whereas the main effect of group was not (P(group) = 0.469). These findings suggest that the relative balance of NK-cell activating and inhibitory signaling evolves differently over time according to IVIG treatment response (Figure 2C).

### 3.5. Early Changes of NKG2A, NKG2D, and NKG2D/NKG2A Ratio After IVIG

**Figure 3:**
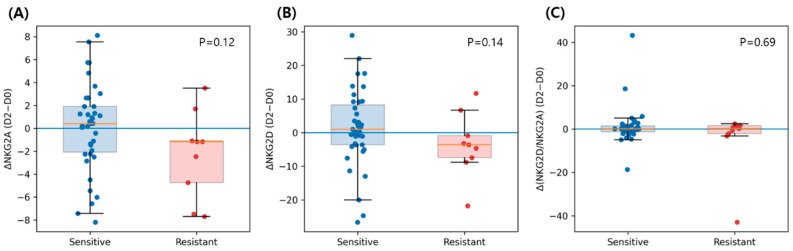
Early changes in NK-cell receptor expression following IVIG treatment. Early changes in receptor expression between baseline (D0) and early post-treatment (D2) were compared between IVIG-sensitive and IVIG-resistant patients. Box plots show (**A**) ΔNKG2A, (**B**) ΔNKG2D, and (**C**) Δ(NKG2D/NKG2A ratio). Each dot represents an individual patient. Blue indicates IVIG-sensitive patients, and red indicates IVIG-resistant patients. *p* values were calculated using the Mann–Whitney U test. Early changes following IVIG treatment show substantial inter-individual variability without clear group separation.

To investigate early immune responses following IVIG administration, changes in receptor expression between baseline (D0) and the early post-treatment timepoint (D2) were examined (Figure 3).

The early change in NKG2A (ΔNKG2A, D2 − D0) tended to differ between groups, with IVIG-sensitive patients showing a slight increase and IVIG-resistant patients showing a tendency toward reduction; however, this difference did not reach statistical significance (*p* = 0.119). Similarly, ΔNKG2D showed a trend toward greater increases in IVIG-sensitive patients compared with IVIG-resistant patients, although this difference was also not statistically significant (*p* = 0.139). Changes in the NKG2D/NKG2A ratio did not differ significantly between groups (*p* = 0.692), although substantial inter-individual variability was observed.

### 3.6. Distribution Across Timepoints

**Figure 4:**
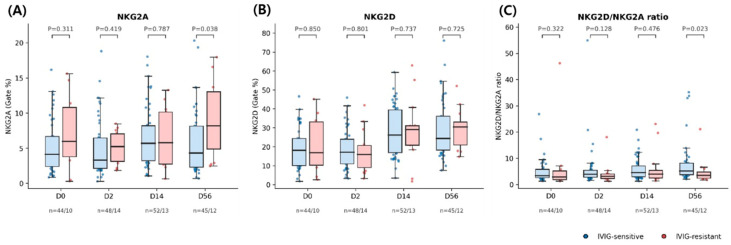
Distribution of NK-cell receptor expression across timepoints according to IVIG response. Box plots illustrate the distribution of (**A**) NKG2A expression, (**B**) NKG2D expression, and (**C**) the NKG2D/NKG2A ratio at each study timepoint (D0, D2, D14, and D56) in IVIG-sensitive and IVIG-resistant patients. Each dot represents an individual patient. *p* values indicate between-group comparisons at each timepoint using the Mann–Whitney U test. A significant divergence between groups is observed at D56, with higher NKG2A expression and a lower NKG2D/NKG2A ratio in IVIG-resistant patients.

To further examine group differences at individual timepoints, the distributions of NKG2A, NKG2D, and the NKG2D/NKG2A ratio were compared between IVIG-sensitive and IVIG-resistant patients at each sampling timepoint (Figure 4). For NKG2A, no significant group differences were observed at baseline (D0), D2, or D14. However, a significant difference emerged at the convalescent phase (D56), with higher NKG2A expression observed among IVIG-resistant patients (*p* = 0.038). In contrast, NKG2D expression did not differ significantly between groups at any timepoint. The NKG2D/NKG2A ratio demonstrated a significant group difference at D56 (*p* = 0.023), suggesting that the balance between activating and inhibitory NK-cell signaling pathways may diverge during the convalescent phase between IVIG-sensitive and IVIG-resistant patients.

### 3.7. Exploratory Clinical Outcome Analysis

Exploratory analyses were performed to examine the association between NK-cell regulatory markers and clinical outcomes, including coronary artery involvement and treatment intensity. No statistically significant associations were identified, likely due to the limited number of coronary events and the low variability in treatment exposure.

## 4. Discussion

Kawasaki disease (KD) is characterized by intense systemic inflammation and complex immune dysregulation involving both innate and adaptive immune pathways. In this prospective longitudinal study, we examined temporal changes in NK-cell regulatory receptor expression in patients with KD and evaluated their relationship with IVIG treatment response. The principal finding of this study is that although baseline NK-cell receptor expression did not differ significantly between IVIG-sensitive and IVIG-resistant patients, the longitudinal trajectory of NK-cell regulatory balance differed between groups. In particular, the NKG2D/NKG2A ratio demonstrated a significant time-by-group interaction, suggesting that the balance between activating and inhibitory NK-cell signaling evolves differently over the disease course according to IVIG responsiveness.

These findings highlight the importance of considering immune kinetics when investigating the immunopathology of KD. Unlike prior studies that have focused on cross-sectional immune profiles at baseline [10,11], our findings highlight that immune dynamics over time—rather than static measurements at presentation—may be more relevant to understanding IVIG responsiveness in KD. Notably, while no significant differences in the NKG2D/NKG2A ratio were observed at baseline, distinct patterns emerged following treatment particularly at the recovery phase (D56) driven by increased NKG2A expression in the IVIG resistant group.

At first glance, the observed decrease in the NKG2D/NKG2A ratio in IVIG-resistant patients may appear counterintuitive, given that NKG2A is an inhibitory receptor. However, increased expression of inhibitory receptors, including NKG2A, has been reported in settings of persistent immune activation and may reflect compensatory or dysregulated immune responses rather than effective immune suppression [12,13]. Accordingly, elevated NKG2A expression in IVIG-resistant patients may indicate altered immune regulation rather than successful suppression of inflammation.

These findings further suggest that IVIG responsiveness may be more closely related to immune regulation during the resolution phase rather than baseline immune activation. The observed increase in inhibitory signaling in resistant patients may reflect delayed or altered resolution of inflammation. This interpretation is consistent with the clinical observation that IVIG-resistant patients had more prolonged inflammation, although these associations should be interpreted with caution. Together, these results support the concept that timely suppression of inflammation may play a critical role in shaping subsequent immune regulatory trajectories in KD, as reflected by the divergent evolution of NK-cell regulatory signaling observed during the recovery phase.

NK-cell activity is regulated by the balance between activating and inhibitory receptor signaling [3,13]. NKG2D is a major activating receptor that promotes cytotoxicity and pro-inflammatory cytokine production upon engagement with stress-induced ligands, whereas NKG2A delivers inhibitory signals through interaction with HLA-E, thereby limiting excessive immune activation [6,7]. The functional state of NK cells is therefore determined not only by the expression of individual receptors but also by the balance between activating and inhibitory pathways [13].

In the present study, both NKG2A and NKG2D showed significant time-dependent changes following IVIG therapy, indicating dynamic modulation of NK-cell signaling. Importantly, the NKG2D/NKG2A ratio demonstrated divergent temporal patterns between IVIG response groups, with a significant time-by-group interaction suggesting differential evolution of immune regulation. These findings further support that differences in treatment response may be related to immune resolution dynamics rather than baseline inflammatory status [14,15].

Baseline NK-cell receptor expression was not significantly associated with IVIG resistance in our cohort. However, given the limited number of IVIG-resistant patients, these findings should be interpreted with caution. In contrast, clear divergence in immune regulatory signaling emerged over time, suggesting that treatment response may be more closely related to longitudinal immune dynamics rather than baseline immune status. This observation is notable because it suggests that the immunologic determinants of treatment responsiveness may emerge during the early post-treatment phase rather than being fully established at disease presentation [4,16,17]. Such a temporal pattern would be consistent with the pleiotropic immunomodulatory effects of IVIG, which include Fc receptor blockade, modulation of cytokine networks, and regulation of innate immune cell activation [5,18,19]. Variability in how these regulatory mechanisms are engaged across patients could contribute to differences in the evolution of NK-cell signaling pathways following treatment [5].

We also examined early changes in receptor expression between baseline and the early post-treatment time point. Although early shifts in NKG2A and NKG2D expression did not differ significantly between IVIG-sensitive and IVIG-resistant patients, substantial inter-individual variability was observed. This heterogeneity likely reflects the complex and multifactorial immune responses induced by IVIG therapy and may explain why early changes did not clearly discriminate between groups, limiting the utility of early biomarkers in predicting treatment response [2,5,18,19].

Baseline clinical and laboratory characteristics in our cohort were broadly consistent with prior studies of IVIG-resistant KD. Patients with IVIG resistance had a longer duration of fever before treatment and exhibited higher levels of systemic inflammatory markers, including CRP and neutrophil-to-lymphocyte ratio [20,21]. However, despite these differences in systemic inflammation, baseline NK-cell immunophenotypes were largely similar between groups, further suggesting that immune regulatory differences may emerge during the subsequent evolution of the inflammatory response [4,22].

An important consideration in interpreting our findings is the clinical relevance of the late divergence in NK-cell regulatory signaling observed at D56. Exploratory analyses did not identify statistically significant associations between NK-cell regulatory markers and these clinical outcomes. However, these analyses were inherently underpowered due to the small number of coronary events and the limited variability in treatment exposure. Therefore, the absence of statistically significant associations should not be interpreted as evidence of no relationship.

The emergence of group differences during the convalescent phase suggests that IVIG-resistant patients may exhibit delayed or altered resolution of immune activation, rather than a distinct immunologic profile at baseline. This pattern may reflect persistent immune dysregulation or compensatory inhibitory signaling following unresolved inflammation. Notably, the fact that these differences become apparent only during recovery highlights the importance of immune dynamics after treatment, rather than baseline immune status alone.

Whether these immune trajectories are associated with clinically meaningful outcomes, including coronary artery abnormalities or treatment burden, remains to be determined in future studies with larger cohorts and integrated longitudinal follow-up.

Several limitations should be considered when interpreting these results. First, the number of IVIG-resistant patients was relatively small, which may have limited statistical power to detect subtle differences in baseline immunophenotypes. Second, this was a single-center observational study, and the findings may not be generalizable to other populations. Third, receptor expression was evaluated as a phenotypic marker, and functional assays of NK-cell activity were not performed, limiting mechanistic interpretation.

Importantly, adjunctive therapies such as corticosteroids and infliximab were administered in a subset of IVIG-resistant patients. These treatments may have influenced NK-cell receptor expression, particularly at later timepoints, and thus could partially contribute to the observed longitudinal differences. Therefore, the extent to which the D56 findings reflect intrinsic disease biology versus treatment effects cannot be fully determined in the present study.

An additional limitation relates to the availability of baseline samples. A subset of patients did not have analyzable baseline immunophenotyping data due to delays in sample processing, particularly during off-hours. This may have resulted in non-random missingness, potentially excluding patients with more acute or severe clinical presentations. Therefore, the possibility of selection bias cannot be excluded, and baseline findings should be interpreted with caution.

Despite these limitations, this study provides longitudinal evidence that NK-cell regulatory signaling undergoes dynamic changes during the course of KD and that the trajectory of these changes may differ according to IVIG treatment response. These findings suggest that time-dependent immune regulatory processes may play an important role in the pathophysiology of KD.

## 5. Conclusions

While baseline NK-cell immunophenotypes were not associated with IVIG response, longitudinal analysis revealed that IVIG-resistant patients exhibited a distinct immune trajectory, characterized by increased NKG2A expression and a lower NKG2D/NKG2A ratio during the convalescent phase. These findings suggest that differences in IVIG responsiveness may be related not to baseline immune status, but to alterations in immune regulatory processes during the resolution phase of inflammation. Therefore, longitudinal immune profiling may provide important insights into the mechanisms underlying treatment response in Kawasaki disease. However, to establish the clinical significance of these findings, larger multicenter studies and analyses incorporating long-term clinical outcomes are required.

## Figures and Tables

**Table 1:** Baseline characteristics of Kawasaki disease patients according to IVIG response.

Variable	Total (*n* = 69)	IVIG Sensitive (*n* = 52)	IVIG Resistant (*n* = 17)	*p* Value
				
Age, years	3.08 [2.04–4.09]	3.11 [2.10–4.08]	2.70 [1.80–5.00]	0.691
Male sex	36 (52.2%)	26 (50.0%)	10 (58.8%)	0.585
Body weight, kg	15.90 [11.80–19.20]	15.15 [11.73–18.52]	16.30 [13.30–20.50]	0.358
Fever duration before IVIG, days	7.00 [5.00–8.00]	6.00 [5.00–7.00]	8.00 [7.00–9.00]	0.009
Rash	58 (84.1%)	44 (84.6%)	14 (82.4%)	1.000
Conjunctivitis	68 (98.6%)	51 (98.1%)	17 (100%)	1.000
Red lips/tongue	54 (78.3%)	39 (75.0%)	15 (88.2%)	0.326
Cervical lymphadenopathy	49 (71.0%)	37 (71.2%)	12 (70.6%)	1.000
Extremity changes	45 (65.2%)	36 (69.2%)	9 (52.9%)	0.251
Complete Kawasaki disease	52 (75.4%)	41 (78.8%)	11 (64.7%)	0.331
Coronary dilatation	4 (5.8%)	2 (3.8%)	2 (11.8%)	0.252
				
WBC, ×10^3^/μL	13.00 [11.20–16.10]	12.80 [11.17–15.72]	13.20 [12.80–16.50]	0.452
Neutrophil, ×10^3^/μL	8.32 [6.82–11.72]	8.13 [6.75–10.47]	9.44 [7.48–14.28]	0.313
Lymphocyte, ×10^3^/μL	2.68 [1.73–3.55]	2.90 [2.04–3.57]	2.26 [1.04–3.43]	0.099
Hemoglobin, g/dL	11.50 [10.70–12.20]	11.50 [10.78–12.20]	11.50 [10.70–12.30]	0.791
Platelet, ×10^3^/μL	382 [310–428]	394 [354–430]	310 [229–419]	0.039
Neutrophil-to-lymphocyte ratio	3.67 [2.08–5.98]	2.60 [1.76–5.38]	4.14 [2.81–10.83]	0.036
CRP, mg/dL	5.47 [3.21–9.38]	4.68 [2.89–8.79]	8.12 [6.72–12.37]	0.014
Sodium, mEq/L	138 [136–139]	138 [136–139]	137.5 [136–140]	0.294
Albumin, g/dL	3.90 [3.60–4.20]	3.90 [3.70–4.20]	3.60 [3.40–4.00]	0.062
AST, U/L	36 [29–84]	34 [27–75]	41 [33–290]	0.092
ALT, U/L	40 [17–151]	27 [17–134]	104 [32–265]	0.027
Total bilirubin, mg/dL	0.37 [0.30–0.54]	0.35 [0.29–0.46]	0.58 [0.32–0.79]	0.022
Troponin-I, ng/mL	0.00 [0.00–0.00]	0.00 [0.00–0.00]	0.00 [0.00–0.01]	0.027
ProBNP, pg/mL	546 [191–1374]	473 [165–1123]	956 [229–3290]	0.044
				
Steroid use	7 (10.1%)	0 (0.0%)	7 (41.2%)	<0.001
Infliximab use	2 (2.9%)	0 (0.0%)	2 (11.8%)	0.058

Values are median [IQR] or n (%). *p* values were calculated using Mann–Whitney U test or Fisher exact test as appropriate.

**Table 2:** Baseline immunophenotype at diagnosis (D0) according to IVIG response.

Immunophenotype Variable	Total (*n* = 57)	IVIG Sensitive (*n* = 44)	IVIG Resistant (*n* = 13)	*p* Value
				
Total NK cells	82.04 (72.30–87.11)	82.62 (76.80–86.83)	71.29 (60.66–87.22)	0.096
CD56^bright^ CD16^+/−^	3.40 (1.78–5.75)	3.27 (1.85–4.65)	3.67 (1.45–7.85)	0.754
CD56^+^ CD16^−^	17.61 (7.86–26.99)	16.44 (7.71–27.09)	22.24 (15.81–26.42)	0.318
CD56^+^ CD16^+^	50.38 (29.53–62.81)	52.69 (34.64–63.67)	36.34 (23.71–52.87)	0.168
CD56^−^ CD16^+^	6.01 (3.95–11.25)	6.37 (4.23–11.49)	5.41 (2.89–6.44)	0.121
				
NKG2D (CD314)	16.97 (10.14–24.37)	18.17 (10.16–24.39)	14.73 (9.08–18.93)	0.512
NKG2A (CD159a)	4.64 (2.43–6.71)	4.13 (2.39–6.67)	5.71 (3.26–8.65)	0.419
NKG2D/NKG2A ratio	3.04 (2.40–5.72)	3.34 (2.59–5.78)	2.51 (1.70–2.89)	0.054
NKp46 (CD335)	13.46 (7.47–20.82)	15.81 (7.44–21.20)	12.90 (7.47–19.70)	0.754
KIR2DL1 (CD158a)	7.66 (4.30–13.37)	8.37 (4.38–13.83)	7.31 (3.27–9.05)	0.291
				
TCRγδ (Gate %)	3.41 (2.49–6.37)	2.99 (2.28–5.58)	4.18 (3.41–6.59)	0.216
Regulatory T cells (CD4^+^CD25^+^Foxp3^+^ Gate %)	0.40 (0.15–0.86)	0.41 (0.17–1.13)	0.21 (0.12–0.78)	0.198

Values are presented as median (IQR). Percentages represent gate-positive proportions within the NK-cell population unless otherwise specified. *p* values were calculated using the Mann–Whitney U test.

## Data Availability

The data presented in this study are available from the corresponding author upon reasonable request. The data are not publicly available due to privacy and ethical restrictions.

## Supplementary Materials

The following supporting information can be downloaded at: https://www.mdpi.com/article/10.3390/children13050635/s1, Table S1: Flow Cytometry Panel and Immunophenotyping Definitions; Table S2: Firth Penalized Logistic Regression Analysis for IVIG Resistance; Table S3: Univariable Firth Penalized Logistic Regression for IVIG Resistance (Baseline D0 Immunophenotype).

## Abbreviations

The following abbreviations are used in this manuscript:

KD	Kawasaki disease
IVIG	Intravenous immunoglobulin
NK	Natural killer
NKG2D	Natural killer group 2 member D
NKG2A	Natural killer group 2 member A
CAL	Coronary artery lesion
CRP	C-reactive protein
ALT	Alanine aminotransferase
NLR	Neutrophil-to-lymphocyte ratio
