# Supplementary material for: Longitudinal Dynamics of NK-Cell Regulatory Signaling and IVIG Response in Kawasaki Disease
Source: Children (Basel). 2026 May 2;13(5):635. doi: 10.3390/children13050635 (PMC13204087; doi:10.3390/children13050635)
Supplement: Supplementary file 1 [file children-13-00635-s001.zip › Supplementary Table S1.pdf]

Supplementary Table S1. Flow Cytometry Panel and Immunophenotyping Definitions

Marker(s)	Cell Population	Functional Category	Definition / Gating Strategy	Measurement
CD56, CD16	Total NK cells	NK subset classification	CD56+ lymphocytes	Absolute number, % of lymphocytes
CD56++ CD16−	CD56bright NK cells	Immature/regulatory NK subset	CD56 high, CD16 negative	Number, % of lymphocytes
CD56+ CD16+	Conventional NK cells	Cytotoxic NK subset	CD56 positive, CD16 positive	Number, % of lymphocytes
CD56− CD16+	CD56dim NK cells	Mature cytotoxic NK subset	CD56 low/negative, CD16 positive	Number, % of lymphocytes
NKG2D	NK receptor	Activating receptor	Expression on gated NK cells	% positive cells, MFI
NKG2A	NK receptor	Inhibitory receptor	Expression on gated NK cells	% positive cells, MFI
NKp46	NK receptor	Activating receptor	Expression on gated NK cells	% positive cells, MFI
KIR2DL1	NK receptor	Inhibitory receptor	Expression on gated NK cells	% positive cells, MFI
TCRγδ	γδ T cells	T-cell subset	CD3+ TCRγδ+ cells	% of lymphocytes
Foxp3	Regulatory T cells	Regulatory marker	CD4+ Foxp3+ T cells	% of CD4+ T cells
