# Supplementary material for: Longitudinal Dynamics of NK-Cell Regulatory Signaling and IVIG Response in Kawasaki Disease
Source: Children (Basel). 2026 May 2;13(5):635. doi: 10.3390/children13050635 (PMC13204087; doi:10.3390/children13050635)
Supplement: Supplementary file 1 [file children-13-00635-s001.zip › Supplementary Table S2.pdf]

Supplementary Table S2. Firth Penalized Logistic Regression Analysis for IVIG Resistance

Variable	Odds Ratio (OR)	95% CI	P Value
Fever duration before IVIG (per 1 day)	1.36	1.03–1.80	0.028
NKG2D/NKG2A ratio (per 1 unit)	0.99	0.90–1.08	0.812

Outcome: IVIG resistance. Model adjusted for fever duration before IVIG and baseline NKG2D/NKG2A ratio (Gate%). Firth penalized logistic regression was used to account for the small number of events. Odds ratios represent the increase in odds of IVIG resistance per 1-unit increase in the predictor.
