# Supplementary material for: Longitudinal Dynamics of NK-Cell Regulatory Signaling and IVIG Response in Kawasaki Disease
Source: Children (Basel). 2026 May 2;13(5):635. doi: 10.3390/children13050635 (PMC13204087; doi:10.3390/children13050635)
Supplement: Supplementary file 1 [file children-13-00635-s001.zip › Supplementary Table S3.pdf]

Supplementary Table S3. Univariable Firth Penalized Logistic Regression for IVIG Resistance (Baseline D0 Immunophenotype)

Category	Predictor (D0)	n	OR (per 1 unit)	95% CI	P value
**NK cell subsets**	CD56brightCD16−/low	57	1.14	0.97–1.34	0.119
	CD56+ CD16−	57	1.01	0.97–1.06	0.561
	CD56+ CD16+	57	0.98	0.95–1.01	0.206
	CD56− CD16+	57	0.91	0.80–1.04	0.177
**NK cell receptors**	NKG2D (CD314)	57	0.99	0.94–1.05	0.702
	NKG2A (CD159a)	57	1.08	0.93–1.25	0.319
	NKG2D/NKG2A ratio	57	1.02	0.95–1.11	0.554
	NKp46 (CD335)	57	0.99	0.93–1.06	0.806
	KIR2DL1 (CD158a)	57	0.95	0.85–1.05	0.288
**T-cell subsets**	TCRγδ	57	1.06	0.92–1.23	0.391
	Foxp3+ Treg	56	0.73	0.35–1.51	0.394

Outcome: IVIG resistance. Firth penalized logistic regression was used to account for the small number of events. Odds ratios (ORs) represent the change in odds of IVIG resistance per 1-unit increase in each variable (percentage point for Gate% variables).
